# Supplementary material for: Impact of a multi-pronged cholera intervention in an endemic setting
Source: PLoS Negl Trop Dis. 2025 Feb 19;19(2):e0012867. doi: 10.1371/journal.pntd.0012867 (PMC11838873; doi:10.1371/journal.pntd.0012867)
Supplement: Attachment — Submitted filename: Reply_reviewer.docx [file pntd.0012867.s002.docx]

We thank the editorial team and the reviewer for their valuable and constructive feedback. It helped improve the manuscript. We provided additional analyses on the expected age distribution of cholera cases based on a reverse catalytic model using our model parameter estimates. However, no external data are currently available to support or disprove our modeling study or any other in this setting. We added a table to help interpret the parameters. We also expanded on some of our assumptions and model structure while contextualizing them with literature on cholera modeling. Finally, we added clarifications in the discussion to nuance our findings and highlight the need for additional research in this setting.

Below is a detailed response to the reviewer’s comments and a description of the changes made in the manuscript to address them when necessary. Every change in the manuscript was highlighted in yellow.

Reviewer 1:

This study uses mathematical modeling to assess the impact of vaccination and WASH improvement campaigns in a cholera endemic setting in eastern DRC, and also uses the model to assess the potential impact of optimal intervention strategies. The model is also used to assess the relative importance of direct transmission vs. indirect transmission via a contaminated environmental reservoir. The estimated impact of the vaccination campaign was relatively small, in large part because the model estimates that a large fraction of the population was immune prior to the outbreak. My main concern is that there is no serological or other evidence that would support this estimate, and if this estimate is off due to factors not considered in the model (heterogeneity in exposure, risk, etc) then the estimates of both the impact of vaccination and the relative importance of the environmental reservoir might be incorrect.

One possible check would be to compare the age-specific population immunity and incidence that would be estimated using a catalytic model with the other model inputs/outputs (FOI and rate of immune waning). How does this compare to estimates of the FOI from a catalytic model fit to the age-specific case data?

We thank the reviewer for this suggestion and we agree for the need for external data to push the discussion further. We want to highlight that we are transparent on the fact that our findings would require serosurveys and/or thorough environmental sampling to be confirmed (see lines 419-420).

To the best of our knowledge, no cholera serosurvey data are currently available in the Democratic Republic of Congo. Some serosurveys are planned or were recently performed by Epicentre/Doctors Without Borders around Lake Upemba, but their results have not been published and the pattern displayed by reported cholera cases in this area differs from what is seen in Kalemie.

Exploring the age specific immunity can then only rely on surveillance data. However, reporting rate is likely heterogeneous across age groups: we would expect higher reporting rate among the age groups most likely to have severe symptoms: children under 5 and elderly people. Thus, fitting a catalytic model to those data would lead to skewed estimates. We want to emphasize that as of today no modeling study, including ours, that finds unexpected results can acquire this kind of external data to support or disprove them in this specific setting; the data simply do not exist.

Despite the important limitation of the use of a catalytic model with data displaying a heterogenous reporting rate we explored what we would expect from a reverse catalytic model based on our model estimates and compared it the age distribution of the suspected cases in the surveillance data.

A reverse catalytic model for a disease with waning immunity is as follows (1):

$$p\left( a \right)=\frac{\phi}{\phi+\delta}(1-e^{-\int_{0}^{a} (\phi+\delta)da})$$

With $p\left( a \right)$ the proportion of individuals who had or have an infection at age a, $\phi$ the force of infection, and $\delta$ the waning immunity rate. If we assume constant $\phi$ and $\delta$ then the reverse catalytic model is as follows:

$$p\left( a \right)=\frac{\phi}{\phi+\delta}(1-e^{-(\phi+\delta)a})$$

Here, we use the average value of the $\phi$ from November 2013 to November 2015 (values are shown in Figure 4C of the manuscript) and the average value of $\delta$ from our model with the best fit (as shown in Table S8 of the Supplementary Information).

We then estimated the expected age distribution of susceptible individuals after reweighting the expected age-specific proportion of susceptibles ($1-p\left( a \right)$) with the age structure of the Congolese population from the Demographic and Health Survey from 2013-2014 (2) and compared it to the age distribution of the suspected cholera cases from November 2013 to February 2016 (Figure 1).

The result is consistent with our expectations given a heterogenous reporting rate: an overrepresentation of children under 5, mainly, and elderly people that could reflect a higher reporting rate in those groups. However, this is insufficient to support or disprove our findings without actual serosurvey data.

Figure 1: Comparison of the age distribution of the susceptible individuals based on a reverse catalytic model after reweighting based on the Congolese age structure (grey bars) and the suspected cholera cases in Kalemie (empty red bars) from November 2013 to February 2016. The red error bars are the 95% confidence intervals.

We added a sentence in the discussion (see lines 420-421) to further highlight that additional research is necessary to confirm our findings and reworded our previous sentence highlighting the need to confirm our estimates.

As an alternative to high levels of complete immunity, there may be varying levels of partial immunity from prior infection/exposure with new exposures leading to asymptomatic infections with immune boosting (e.g., King et al. 2008 Nature) and lower levels of shedding. It might be useful to model this possibility, with vaccine providing additional protection beyond naturally-derived immunity. In lines 372-377 you mention the duration of immunity from challenge studies, but the infection of naive individuals in these studies might not represent the infections or "incidence rate" observed in the cited Bangladesh study of vibriocidal titers.

On immunity in asymptomatic cases:

Regarding the findings on shorter immunity among asymptomatic cases from King et al, they are interesting but unconventional given the very short duration they suggest. Their model with the best fit is backed-up by little external data regarding short-lived immunity among asymptomatic cases on the scale of weeks. As far as we are aware, the observational study done by Woodward et al is the only one with information on mild/asymptomatic cholera cases (3). It included 7 cholera cases with mild or no symptoms during their first infection out of 14 cases who had 2 infections during the study. The interval between the 2 infections ranged from 1.5 to 49 months with an average of 20.5 months in those 7 cases.

This study, although valuable, gathered too few data to confirm or disprove the findings by King et al, but the observed interval between consecutive infections among cases with mild or asymptomatic symptoms appeared much longer than weeks for 6/7 participants (with ~6 weeks, 1.5 months, for the 7^th^ participant only). Interestingly, no experimental or observational data have further supported the King study’s finding since its publication in 2008. Overall, the data regarding cholera immunity is limited and rely on a small number of studies with small sample sizes: very little is known on the immunity duration among asymptomatic cases (4).

We agree that a period of shorter immunity for asymptomatic cases is a likely possibility. However, adding a compartment with asymptomatic infections would require making assumptions regarding waning immunity for asymptomatic cases with very little data available to support them. We do not think this would be a reasonable approach given the absence of information on asymptomatic cases in the surveillance data used to fit our model.

We added a sentence in the discussion (see lines 444-446) to highlight that our estimate of the duration of immunity reflects a simplification and an average over several different dynamics depending on the infection severity.

On the varying levels of protection for immune individuals receiving a vaccine:

We also agree that similar immunity for successfully vaccinated and infected individuals is a simplification, and we acknowledge this in the discussion (see lines 450-452). Compartmental models, the most common modeling framework for cholera (5), often apply such simplifications to avoid adding unnecessary complexity given the research question and the imperfect data used to fit them. In our model, the short study period removes the cumulative problems associated with this simplification.

Considering more complex transmission patterns with varying transmissibility may also reflect reality more closely, as would including various levels of susceptibility, or considering the origin of migrating people. However, a model should only be as complex as necessary given its goal.

No model claims to accurately capture all aspects of cholera. The trade-off for simplifications, specifically a smaller number of required assumptions and parameters, is to increase complexity where it matters the most given the research question. Imperfect data, like the ones typically available from non-experimental settings, constrain the complexity of the model and force us to sparingly increase complexity on the aspects that are most interesting to us. Our aim was to explore the relative contribution of environmental-based and interhuman transmission. Our model incorporated a fair amount of complexity on that topic and provided valuable and novel insights that justified the necessary simplifications. While we are interested in exploring additional scenarios to learn more about the system, adding complexity on two types of immunity, infection- or vaccine-derived, would add little value to our findings given our study duration (118 weeks) as we emphasized in the discussion (see lines 452-457). We agree that this would be a valuable addition to the model structure for a longer time series though and added a sentence on this limitation lines(see lines 457-459).

On the varying levels of protection between naïve and frequently exposed cases:

We agree that the average immunity duration of naïve cases is most likely not a direct reflection of the pattern that we can expect from frequently exposed cases. However, the findings of observational studies performed in endemic areas where participants had likely been exposed previously, Bangladesh and East Pakistan, were reasonably consistent with challenge studies including naïve participants (4).

Although, as mentioned previously, current knowledge relies on a small number of studies and data points, this aspect of infection is better documented than immunity among asymptomatic cases and highlights that simplifying immunity on this specific aspect is reasonable and realistic given our study duration.

Having a single environmental reservoir for the entire population might also overestimate its contribution to transmission. Metapopulation modeling was mentioned in the introduction, so it would be useful to understand if was considered here and if not, why not?

Metapopulation models have previously been used for cholera and they typically use settlements or administrative units as patches with a common environmental exposure at patch level (6–8).

One reason for using a common environmental reservoir is that detailed knowledge on where or at least tentative explanations for how environmental exposure varies spatially would be necessary to fit a spatially explicit metapopulation model for a single settlement. As far as we know, nobody has such information at the resolution that would be necessary for Kalemie. Further, fitting a spatially explicit model would also suffer from a potentially spatially heterogeneous reporting rate in the surveillance data.

To the best of our knowledge, SIRB models used for cholera have all had a common environmental reservoir, whether it is theoretical studies like Codeço et al or modeling studies using surveillance data like Lemaitre et al (9,10).

The conjunction of the economic importance of Lake Tanganyika for Kalemie, with strong fishing activities, and the high percentage, 35.3%, of households whose main source of drinking water is surface water in this Province, Tanganyika (2), suggests that this simplification is reasonable. The endemicity of cholera in the African Great Lakes region (11) and the occurrence of cholera hotspots in DRC along its lakes (12) further support it. There is also microbiological evidence for the presence of toxigenic *vibrio cholerae* in the lake (13).

We added a sentence in the discussion (see lines 466-468) to nuance our findings and highlight that they reflect an “average” situation, potentially hiding spatial heterogeneity in environmental exposure.

Particularly given that data from this outbreak in Kalemie was previously used to show that risk of infection was higher within 200m of a previous case (Azman et al. 2018 JID), suggesting that the environmental risk varies spatially (or direct transmission is more common than estimated here).

The analysis performed by Azman et al is interesting with important limitations. Specifically, the tau statistics is descriptive and cannot assess the environmental contribution to cholera transmission on its own for the following reasons:

- Pollington et al have also demonstrated that typical use of the tau statistic in the literature, including in this study, tends to be biased and under-estimates the size of the clusters (14). They cite the study from Azman et al, among others, as a study misusing the tau statistic in the way they assess the clustering endpoint and provide methods to attempt to correct such biases.
- The study assumes that infection is by default acquired at home. We agree that it is likely a common occurrence but not the only possibility.
- The tau statistics measure the tendency of pairs of cases to cluster. There is no certainty that the “primary case” in the considered pairs is actually the primary case in a context of strong under-reporting. Whether the increased risk is the consequence of the presence of the “primary case” or several other cases in the area that occurred just one day before and were not reported cannot be tested.
- The tau statistic is descriptive and does not allow us to disentangle increased localized risk because of a common exposure like a contaminated water reservoir or more frequent direct contacts with infected individuals.

We do not think that assuming a potentially common environmental exposure for a small city stretched on the shore of a lake with low access to clean water and high prevalence of diarrheal diseases is unreasonable. The literature provides multiple examples of such assumptions. We agree that the environmental exposure likely varies spatially and that our findings provide an “averaged” picture of what is most likely a heterogeneous situation with very high environmental exposure in some places and not in others. We added clarifications in the discussion (see lines 466-470).

From line 130 ("...northern part of the city laying pipes...") it sounds like the WASH intervention was more narrowly geographically targeted. Is this assumption correct, and if so could the model be subdivided in a way that would incorporate this difference?

Some aspects of the WASH intervention were narrowly geographically targeted and some were widespread. Laying pipes was only in one part of the city, but the distribution of water filters and the installation of water pumps and some occasional chlorination activities were done over a large area that are impossible to clearly delineate (water filters were used at home and probably beyond the original areas).

We agree that incorporating this targeted characteristic, as we did for the vaccination campaign, would be valuable. However, incorporating the targeted nature of laying pipes would require disentangling its effect from the effects of the other components of the WASH intervention, which is not possible given the information available. Dedicated surveys were done to precisely spatially characterize the uptake of the vaccination campaign. A similar detailed mapping and summary of activities for the WASH intervention was never planned or executed.

We added a sentence in the discussion (see lines 461-463) to further emphasize that our estimate of the impact relies on a simple characterization and that more detailed information could have allowed us to refine our estimates.

Minor comments

1.It would be helpful to have a table with parameter descriptions in the main text for easy reference

We agree and have added a similar table to the one in the Supplementary Information with parameter interpretation in the manuscript.

2. In the Results and/or 1st paragraph of the Intro it would be helpful to also present the cases averted by vaccination and WASH as a % of total cases in the absence of either intervention. The raw numbers seem high relative to the number of observed cases, but I assume that is because you also estimate a large fraction of infections/cases were unreported?

We added the relative reduction in the results (see lines 292-295).

Yes, the model estimates a large fraction of unreported cases with an average reporting rate of 1.3%.

3. While the counterfactual simulations of incidence under interventions that completely prevent contamination or exposure of the environment are nice examples of the potential power of improving access to clean water and sanitation, real-world WASH interventions rarely have impacts as large as those estimated here. It might be useful to also see the impact of interventions with reductions in contamination and/or exposure that are reflectively of some previous interventions.

We agree it would be useful to explore more nuanced scenarios. However, that work is beyond the scope of this study. Our goal was to further illustrate the environmental contribution to transmission, and it is better achieved by simply removing it. Additionally, cholera is no longer a public health problem in high income countries, partially because they implemented and maintained such WASH interventions (15). As an example, the United Kingdom, which was hit by large cholera outbreaks during previous pandemics, is not at risk for such outbreaks nowadays despite still importing cases (23 imported cholera in 2022 (16)) partially because they have the necessary sanitation infrastructure to ensure that its almost totally susceptible population will not be exposed to or contaminate its environment. We feel this work builds a solid foundation for future work to investigate more subtle WASH intervention scenarios, similar to what you have suggested.

References:

1.	Pomeroy LW, Bjørnstad ON, Kim H, Jumbo SD, Abdoulkadiri S, Garabed R. Serotype-Specific Transmission and Waning Immunity of Endemic Foot-and-Mouth Disease Virus in Cameroon. PLOS ONE. 2015 Sep 1;10(9):e0136642.

2.	MPSMRM/Congo M du P et S de la M en œuvre de la R de la M, MSP/Congo M de la SP, International ICF. République Démocratique du Congo Enquête Démographique et de Santé (EDS-RDC) 2013-2014. 2014 Sep 1 [cited 2024 Dec 10]; Available from: https://dhsprogram.com/publications/publication-FR300-DHS-Final-Reports.cfm

3.	Woodward WE. Cholera Reinfection in Man. The Journal of Infectious Diseases. 1971 Jan 1;123(1):61–6.

4.	Leung T, Matrajt L. Protection afforded by previous Vibrio cholerae infection against subsequent disease and infection: A review. PLOS Neglected Tropical Diseases. 2021 May 20;15(5):e0009383.

5.	Anteneh LM, Lokonon BE, Kakaï RG. Modelling techniques in cholera epidemiology: A systematic and critical review. Mathematical Biosciences. 2024 Jul 1;373:109210.

6.	Njagarah JBH, Nyabadza F. A metapopulation model for cholera transmission dynamics between communities linked by migration. Applied Mathematics and Computation. 2014 Aug 15;241:317–31.

7.	Senapat A, Sardar T, Chattopadhyay J. A Cholera Metapopulation Model Interlinking Migration with Intervention Strategies - a Case Study of Zimbabwe (2008-2009). J Biol Syst. 2019 Jun;27(2):185–223.

8.	Bertuzzo E, Azaele S, Maritan A, Gatto M, Rodriguez-Iturbe I, Rinaldo A. On the space-time evolution of a cholera epidemic. Water Resources Research [Internet]. 2008 [cited 2022 Mar 8];44(1). Available from: http://onlinelibrary.wiley.com/doi/abs/10.1029/2007WR006211

9.	Codeço CT. Endemic and epidemic dynamics of cholera: the role of the aquatic reservoir. BMC Infectious Diseases. 2001 Feb 2;1(1):1.

10.	Lemaitre J, Pasetto D, Perez-Saez J, Sciarra C, Wamala JF, Rinaldo A. Rainfall as a driver of epidemic cholera: Comparative model assessments of the effect of intra-seasonal precipitation events. Acta Tropica. 2019 Feb 1;190:235–43.

11.	Bompangue Nkoko D, Giraudoux P, Plisnier PD, Tinda AM, Piarroux M, Sudre B, et al. Dynamics of cholera outbreaks in Great Lakes region of Africa, 1978-2008. Emerging Infect Dis. 2011 Nov;17(11):2026–34.

12.	Taty N, Bompangue D, Muyembe J, Richmond N. Spatiotemporal dynamics of cholera in the Democratic Republic of the Congo before and during the implementation of the Multisectoral Cholera Elimination Plan: A cross-sectional study from 2000-2021. 2022.

13.	Hounmanou YMG, Njamkepo E, Rauzier J, Gallandat K, Jeandron A, Kamwiziku G, et al. Genomic Microevolution of Vibrio cholerae O1, Lake Tanganyika Basin, Africa - Volume 29, Number 1—January 2023 - Emerging Infectious Diseases journal - CDC. [cited 2023 Apr 13]; Available from: https://wwwnc.cdc.gov/eid/article/29/1/22-0641_article

14.	Pollington TM, Tildesley MJ, Hollingsworth TD, Chapman LAC. Developments in statistical inference when assessing spatiotemporal disease clustering with the tau statistic. Spatial Statistics. 2021 Apr 1;42:100438.

15.	Luby SP, Davis J, Brown RR, Gorelick SM, Wong THF. Broad approaches to cholera control in Asia: Water, sanitation and handwashing. Vaccine. 2020 Feb 29;38:A110–7.

16.	Santé WHO= O mondiale de la. Cholera, 2022 – Choléra, 2022. Weekly Epidemiological Record = Relevé épidémiologique hebdomadaire. 2023 Sep 22;98(38):431–43.
